# Haematological and biochemical analysis of blood samples from early and late stage breast cancer patients in India

**DOI:** 10.6026/97320630019806

**Published:** 2023-07-31

**Authors:** Smriti Shreya, Anusmita Shekher, Puneet Puneet, Shyam Babu Prasad, Buddhi Prakash Jain

**Affiliations:** Department of Zoology, Mahatma Gandhi Central University, Motihari, Bihar, India; Department of General Surgery, Institute of Medical Sciences, Banaras Hindu University Varanasi, Uttar Pradesh; Department of Biochemistry, Institute of Sciences, Banaras Hindu University Varanasi, Uttar Pradesh; Gene Expression and Signaling Lab, Department of Zoology, Mahatma Gandhi Central University, Motihari, Bihar, India

**Keywords:** Breast cancer, haematology, tumor, urea, haemoglobin

## Abstract

Breast cancer is the most prevalent cancer with the maximum number of cases worldwide. Early diagnosis of the cancer is necessary for an effective treatment plan. Due to a lack of awareness, diagnosis of breast cancer at an early stage is difficult. The present study aims to evaluate and compare the haematological and biochemical profiles of the early and late-stage breast cancer patient's data records. A retrospective cohort study was conducted on 56 breast cancer patients at the Institute of Medical Sciences, Banaras Hindu University India. Patient data records were obtained and haematological and biochemical parameters were arranged on an Excel sheet and analyzed. Random blood sugar (RBS), alkaline phosphates (ALP) levels, and urea levels were significantly high in patients with late-stage breast cancer (Tumor stage III and IV). At the advanced stage of breast cancer hemoglobin level falls and patients became anemic. Further large-scale studies with a greater number of patient data can help establish these parameters individually or in combination as prognostic and diagnostic markers in breast cancer staging.

## Background:

Cancer is a disease where cells divide abnormally and have the potential to invade and spread to other parts of the body. Breast cancer is one of the most common cancers prevalent worldwide. In Breast cancer, a tumor is usually formed inside either the ducts (invasive ductal carcinoma) or lobules (invasive lobular carcinoma) or in other cells or tissues inside the breast. It is one of the leading causes of death in women, especially in developing countries. Cases of breast cancer in males are rare. According to World Health Organization (WHO) data, 2.26 million cases were reported Breast Cancer in 2020 while 685000 deaths occurred by Breast cancer [1]. One in every eight women is likely to get affected by breast cancer. Breast cancer can be spread through blood vessels or lymph vessels. It can also spread to the liver, lungs, also in bones, and brain. Breast cancer can be classified based on the growth, size, and stage of the tumor. The expressions of various genes are used in the prognosis of breast cancer. The tumor can be tested for expression of estrogenic receptors (ER Receptors), progestogen receptors (PR), and HER2/neu proteins. Breast cancer comes under the flagship of interaction between genomic constituents as well as environmental factors. Some risk factors also include hormones, lifestyle, obesity, radiation exposure, early menstruation or late menopause, alcohol, smoking, and late pregnancy. It has also been found that mutations in genes like BRCA1, and BRCA2 cause the risk of breast cancer from parents to their offspring [2,3]. TNM classification is widely used for staging breast cancers which includes tumor size, lymph node involvement, and metastasis [3]. Complete blood count and biochemical analysis of blood samples are prerequisites for investigating breast cancer in suspect patients. Any kind of damage or severity will change hematologic and biochemical parameters in diseased conditions. Hence, complete Blood count, biochemical parameters, RBCs counts, Hb Level, TLC count, DLC count, platelets count, random blood sugar level, and liver function tests which include serum glutamate oxaloacetate transaminase SGOT, serum glutamate pyruvate transaminase (SGPT), STB/SDB, Alkaline phosphate, creatinine, sodium, potassium, urea has been taken in our study to analyze the haematological analysis with breast cancer stage. These are parameters that help in diagnosing other diseases. Liver function tests measure chemicals like liver transaminase, albumin, Serum proteins, bilirubin, alkaline phosphatase, and blood glucose levels. Kidney function tests (KFT) include blood urea, creatinine, calcium, sodium, and potassium. The rate of survival increases with early diagnosis. However most of the patients are diagnosed at advanced stages which gets difficult to be treated. Therefore, it is of interest to analyse the various haematological and biochemical parameters in the blood of early and late-stage breast cancer patients. Random blood sugar, urea level, alkaline phosphatase, and haemoglobin levels were some of the parameters which were significantly different in the early and advanced stages of breast cancer. Further study with a large number of patient data can be useful to establish these blood parameters, and liver and renal function tests as diagnostic markers for the prediction of the early and late stages of the disease.

## Methods:

This comprises a retrospective cohort study for 2 years. The study started after obtaining approval from the ethical committee from IMS, BHU. It is located around 314 km from Lucknow, the capital city of Uttar Pradesh. All cancer patients attended the surgical oncology unit of Sir Sundarlal Hospital, BHU. Patient data were obtained from the hospital record. The study group contains 56 patients which included female patients diagnosed with breast cancer. The medical record number (MRN) of the patient had been collected. The files of the cancer patients were obtained by using the data collection form. Data included age, hematologic and biochemical profile along with demography as age, past surgical history from medical records of the patients. The data were arranged in Excel and were filtered according to age wise, stage-wise, and various haematological and biochemical parameters. Analysis of these parameters along with the staging of the breast cancer was done before starting any treatment.

## Results:

The demographic profiling of various patients in the present study is summarized in Table 1. Out of 56 patients in the study, 33 are below the age of 50, and 23 patients are above the age of 50. Among the total number of Breast cancer patients in the study 30% population lies in the age group of 50-60 years and 30% of the population lies within the age of 40-50 years. Patient data were further classified based on clinical characteristics and summarized in Table 2. T0, T1, and T2 stages were considered early-stage breast cancer and T3 and above are placed in late-stage breast cancer. The data collected suggested that only 32% of the total samples have been diagnosed with T0, T1, and T2 stages. 32% of the samples were under stage T3 and 36% of the samples lie under the T4 tumor stage. The data suggest that early diagnosis of breast cancer is not predictive; it might be due to lacking early biomarkers or awareness among the people.

Many haematological and biochemical parameters as haemoglobin, total leukocytes count, differential leucocytes count, random blood sugar, SGPT & SGOT, Serum total bilirubin, serum direct bilirubin, ALKP, total protein, albumin, creatinine, sodium, potassium, urea, platelets, calcium were taken into study. Random blood sugar, urea level, haemoglobin, and alkaline phosphatase level were found to be significantly different in the serum of early and late-stage breast cancer patients. The normal range of RBS lies within 80-140 mg/dL. Out of 56 patients, 20 patients had high RBS. It has also been found that 85% of the high RBS was reported in late-stage breast cancer patients while only 15% of the population has increased RBS in the early stage suggesting that elevated RBS is associated with high-grade tumors. Earlier studies reported the relationship between random blood sugar and cancer. An increase in insulin causes an increase in cell growth and cell proliferation. High glucose level enhances cell proliferation and hence enhances invasive and migrative capacities. High RBS inhibits angiotensin levels which also act as an additional factor for cancer cell proliferation [5]. Patients with elevated RBS are found to have less survival rate. Another parameter is the alkaline phosphatase (ALP) level in the blood which is a marker to test liver function. ALP is a nonspecific enzyme whose serum level suggests the activity of other iso-enzymes which are found in the intestine, bone, liver, and kidney [2,6]. The normal range of ALP in the blood is 40-125 mg/dL. Out of the total patients in the study, 31 patients have elevated ALP which contributes that 41% of the total patients having early-stage breast cancer while 59% of the total patients have advance stage cancer. It has already been studied that high ALP predicts metastasis in bone and liver. The result here showed elevated ALP in advanced-stage breast cancer patients. The progressive increase in ALP level with the stage progression (shown in Table 1) is a clear indication of metastasis. It can be used as a cost-effective diagnostic marker if studied further with a greater number of patients' data [7]. The anaemic condition can occur due to haemolysis, disturbed RBC production, or blood loss [8]. From the data collected, it has also been found that 30 patients were anaemic 56. 84% of the anaemic patients were in the late stage while 16% were in the early stage. This observation pointed out that maybe with the advancement of the disease, the patients become more anaemic and their Hb level falls. This can be another haematological marker for the prediction of the stage and progression of the disease. Most of the anaemic patients were young. It has been studied that metastasis in breast cancer suppresses haematopoiesis. Urea is an excretory waste of the body and is formed in the liver by the ornithine cycle. Urea is excreted out by urine as a renal function. Blood urea level is an important indicator of kidney function. The normal range of urea in blood is 20-40 mg/dl. It has been found that 70% of the patients have advanced breast cancer and 29.62% of the patients with elevated Urea have been found in the early stage. 75% of the elevated total protein is found which comprises the advanced stage of breast cancer. Significant correlations have been found between total proteins and serum urea levels with the advancement of cancer. Elevated urea level is also linked with renal cell carcinoma [9].

## Discussion:

Since breast cancer is found to be a highly heterogenous group of diseases. Biochemical analysis of blood measures the amount of chemical substance which is released by the body tissues produced during the metabolism of a particular substance. It gives important information about the function of the liver, kidney, and other organs. In this short study, it was found that few of the parameters as random blood sugar (RBS), alkaline phosphatase (ALP) level, urea level, and hemoglobin level were notably different in the patients of early-stage and late-stage breast cancer. RBS, ALP, and urea levels increased with the advancement of the disease and Hb level falls with disease progression. Further study with a large number of patient data can confirm these biochemical parameters in the prediction of stages of breast cancer. Also, a combination of these analyses can be helpful for the prognosis of the diseases.

## Conclusion:

This study focused on assessing the haematological and biochemical analysis of blood samples from early and late stage of breast cancer. Alteration in levels of biochemical components of blood affects various organs and analyzing these parameters can be helpful in providing valuable insight for disease progression and their treatment options. However, additional studies with larger sample sizes need to be done for consistent findings and future research.

## Figures and Tables

**Table 1:** Demographic Characteristics of the Patients

**Age**	**Number of Patients**
21-30 Years	4
31-40 years	12
41-50 years	17
51-60 years	17
61-70 years	5
71-80 years	1

**Table 2:** Clinical characteristics and hematological/Biochemical parameters:

**Tumor Stage**	**Number of Patients**	**Number of Patients with higher RBS**	**Number of Patients with higher ALP level**	**Number of Patients with low Hb level (anemic)**	**Number of Patients with high Urea level (uremic)**
T0	3	3	3	2	0
T1	1	1	1	0	1
T2	14	7	7	4	7
T3	18	13	13	13	8
T4	20	15	15	11	11
